# Receive, Sustain, and Flow: A simple heuristic for facilitating the identification and treatment of critically ill patients during their hospital journeys

**DOI:** 10.7189/jogh.13.04139

**Published:** 2023-12-22

**Authors:** Jacob McKnight, Tamara Mulenga Willows, Jacquie Oliwa, Onesmus Onyango, Elibariki Mkumbo, John Maiba, Karima Khalid, Carl Otto Schell, Tim Baker, Mike English

**Affiliations:** 1 Health Systems Collaborative, University of Oxford, Oxford, England, UK; 2 Health Services Unit, KEMRI-Wellcome Trust Research Programme, Nairobi, Kenya; 3 Department of Paediatrics & Child Health, University of Nairobi, Nairobi, Kenya; 4 Department of Health Systems, Ifakara Health Institute, Dar es Salaam, Tanzania; 5 Department of Anaesthesia, Muhimbili University of Health and Allied Sciences, Dar es Salaam, Tanzania; 6 Department of Global Public Health, Karolinska Institutet, Stockholm, Sweden; 7 Centre for Clinical Research Sörmland, Uppsala University, Eskilstuna, Sweden; 8 Department of Medicine Nyköping Hospital, Nyköping, Sweden; 9 Department of Clinical Research, London School of Hygiene and Tropical Medicine, London, England, UK

## Abstract

**Background:**

Hospital patients can become critically ill anywhere in a hospital but their survival is affected by problems of identification and adequate, timely, treatment. This is issue of particular concern in lower middle-income countries’ (LMICs) hospitals where specialised units are scarce and severely under-resourced. “Cross-sectional” approaches to improving narrow, specific aspects of care will not attend to issues that affect patients’ care across the length of their experience. A simpler approach to understanding key issues across the “hospital journey” could help to deliver life-saving treatments to those patients who need it, wherever they are in the facility.

**Methods:**

We carried out 31 narrative interviews with frontline health workers in five Kenyan and five Tanzanian hospitals from November 2020 to December 2021 during the COVID-19 pandemic and analysed using a thematic analysis approach. We also followed 12 patient hospital journeys, through the course of treatment of very sick patients admitted to the hospitals we studied.

**Results:**

Our research explores gaps in hospital systems that result in lapses in effective, continuous care across the hospital journeys of patients in Tanzania and Kenya. We organise these factors according to the Systems Engineering Initiative for Patient Safety (SEIPS) approach to patient safety, which we extend to explore how these issues affect patients across the course of care. We discern three repeating, recursive phases we term Receive, Sustain, and Flow. We use this heuristic to show how gaps and weaknesses in service provision affect critically ill patients’ hospital journeys.

**Conclusion:**

Receive, Sustain, and Flow offers a heuristic for hospital management to identify and ameliorate limitations in human and technical resources for the care of the critically ill.

Critically ill patients are defined as those in “a state of ill health with vital organ dysfunction, a high risk of imminent death if care is not provided and the potential for reversibility” [1]. In response to the COVID-19 pandemic, many countries increased their intensive care unit (ICU) capacity but a new approach focuses on provision beyond critical care departments [2]. Essential Emergency and Critical Care (EECC) is conceptualised as the effective and feasible identification and treatment of critically patients in any setting [3,4]. Essential Emergency and Critical Care is cost-effective and designed to meet a large existing patient safety gap in health systems around the world challenging us to think beyond interventions that target specific areas of the hospital or particular diseases [5-7].

Strategies to improve services for critically ill patients in low and middle-income countries (LMICs) with few resources often only address specific diseases or specialties [8-11]. However, efforts concentrated in one department are unlikely to influence the quality of care delivered once they move to other departments [12]. This is recognised in high income settings as a problem of integration across the hospital journey and is the subject of quality improvement efforts [13-15]. However, many approaches do not deal with the “problem of many hands” which results in a lack of “ownership” of whole patient experiences and the problems that result from moving between health care providers [11,16].

We aimed to examine the hospital journey of critically ill patients in LMIC hospitals in an approach drawing on frameworks such as by System Engineering Imitative for Patient Safety 3 (SEIPS3) (Appendix 1 in the **Online Supplementary Document**) [17]. Systems Engineering Initiative for Patient Safety is framework that approaches health care safety and quality from a systems perspective, examining processes, outputs and outcomes within complex systems. We sought a practical, generalisable heuristic that could guide the thinking of hospital managers in LMIC who often do not have the support of improvement teams with dedicated time and resources. We identify three generic phases across hospital journeys. The Receive, Sustain, and Flow heuristic provides a common language to describe the work of the hospital into which opportunities for identification and treatment of the critically ill can be embedded.

## METHODS

### Study setting

Our study took place in five Kenyan and five Tanzanian hospitals from November 2020 to December 2021, during the COVID-19 pandemic. We purposefully selected ten facilities, five per country, in different regions across both countries to include a mix of district, regional and tertiary hospitals.

### Data collection

Our study used in-depth interviews (IDI) with hospital staff and observations of hospital journeys to examine how care for critically ill patients is delivered in Kenyan and Tanzanian facilities [18-20]. The iterative combination of the two approaches helped us develop a multi-faceted understanding of care.

#### In-depth interviews (IDI)

We purposively identified staff interviewees to ensure diversity in hospital roles after rapport was established during an earlier phase of the study. In-depth interviews were conducted in English and Swahili with 31 of 33 invited respondents in the 10 study hospitals including 20 medical and 11 nursing personnel. Interviews were conducted by phone (n = 15), in person (n = 15) or Microsoft Teams (n = 1) at times that aimed to minimise disruption to services. All interviews were audio recorded then transferred onto password protected cloud storage databases. A detailed outline of participant demographics is included in Appendix 2 in the **Online Supplementary Document**.

Once interviews were transcribed verbatim, all audio recordings were deleted. Participants were given an opportunity to provide feedback on the analysis of transcripts at data verification meetings conducted at facilities.

#### Hospital journeys

Our hospital journey approach focused on the care of single patients and the conditions within which this care was delivered. We used a purposive sampling approach as IDIs were completed to identify patients from the emergency department or wards who nursing staff thought were potentially critically ill [21]. Researchers observed care provided to patients, interactions between staff regarding critically ill patients and took note of time taken to initiate interventions and monitoring for the observed patient. We did not interview patients or staff due to the critical nature of their condition. We also alerted staff to signs of deterioration in patients left unattended or if these signs were missed by staff to prevent avoidable harm.

We conducted a total of 12 patient journeys, six in Kenya and six in Tanzania across four hospitals. We could not conduct a patient journey at every hospital included in our study due to heightened COVID-19 restrictions.

All the observations and reflections were recorded and later transcribed and stored on a password protected cloud storage database. We did not keep separate field notes.

### Data analysis

#### In-depth interviews (IDI)

Five of the authors participated in coding transcripts and used NVIVO® to manage the data. We developed a code book reflecting both inductive themes and the EECC themes. After all interviews were conducted, we used Gioia tables to develop higher order thematic groups and links to established theories (Gioia et al., 2013). Within our Gioia table we used deductive themes taken from SEIPS: tools and technology; task; organisation; person; and physical environment, to further categorise our themes according to which part of the hospital system they might affect. Participants provided feedback to the team at data verification meetings held at facilities but this feedback did not result in changes being made to the data as they were in agreement with the findings presented.

#### Hospital journeys

We used the observations and reflections made in the field to write the hospital journeys presented below through a process of narrative synthesis [22,23]. This process allowed us to identify topics that would be discussed in greater depth in IDIs. Our narrative synthesis involved using the contextual knowledge of our Tanzanian and Kenyan research assistants as well as the clinical knowledge of our team members to build an understanding of what transpired in each hospital journey. We undertook this process before completing our IDI data collection and used the information to guide our analysis of completed IDIs.

### Ethics statement

We were granted ethical approval from the KEMRI Scientific and Ethical Review Committee (SERU Number 4085), Ifakara Health Institute (IHI/IRB/No:42-2020), the Tanzanian National Institute for Medical Research NIMR/HQ/R.8c/Vol.1/1881 and London School of Hygiene and Tropical Medicine (Ref: 22575 and 22 866). Audio recordings of interviews were deleted once interviews were transcribed. Participants gave consent for use of anonymised quotes from interviews.

## RESULTS

**Figure 1:**
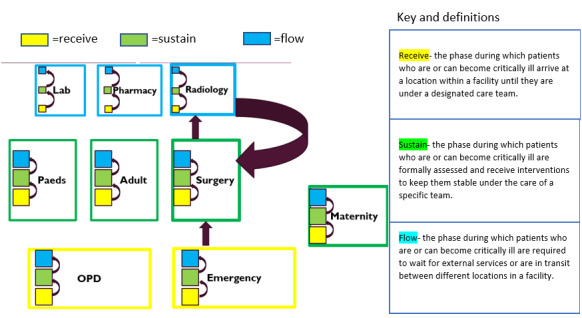
Phases of care – Receive, Sustain, and Flow are recursive and occur at the level of the facility, but also at the level of the ward or unit. Arrows in our diagram illustrate the movement of a patient within and between departments during a hospital journey.

The findings are organised into three categories and based on a conceptual framework developed after deductive and inductive analysis: “Receive”, “Sustain, and “Flow” (RSF). The development of RSF was prompted by our analysis of the patient journeys and situating our IDI findings within the patient journey. **Figure 1** provides definitions for Receive, Sustain, and Flow and illustrates how our concept relates to the journey of critically ill patients in hospitals as each department aligns with either receive, sustain or flow. Furthermore, within each department, we convey how a patient can move within these phases at a sub departmental level while simultaneously moving through these phases at a departmental level.

**Figure 2:**
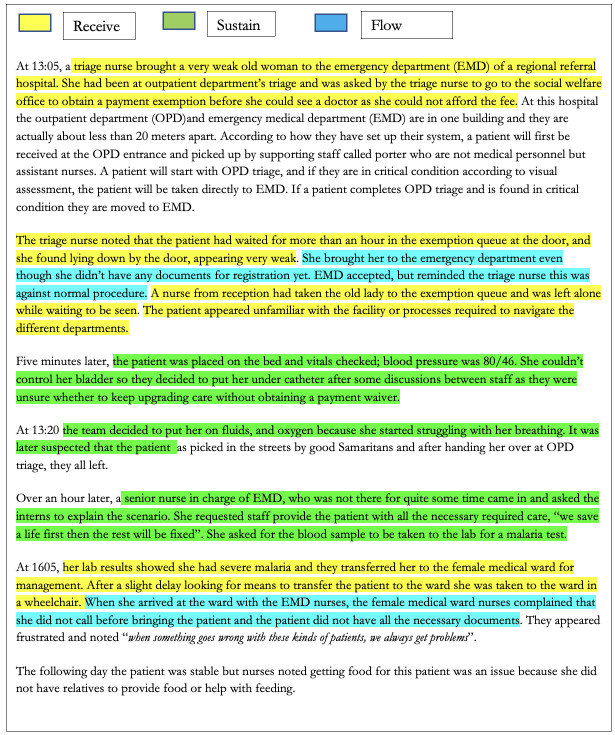
Facility C Tanzania. A hospital journey of an elderly women who becomes critically ill. This hospital journey illustrates the delays in initiating and maintaining care that can occur when payment systems are unable to make exceptions for critically ill patients.

**Figure 2** and Appendix 3 in the **Online Supplementary Document** illustrate how Receive, Sustain, and Flow reoccurred in the hospital journeys conducted in Kenyan and Tanzanian hospitals. Appendix 3 in the **Online Supplementary Document** illustrates how irrespective of the age of patients or where their critically ill state is diagnosed, issues pertaining to receipt, sustain, and flow reoccur.

Receipt is prolonged and subsequently, investigations required during the Sustain phase are also delayed contributing toward deterioration in this patient. During observed periods of Flow, this patient was unmonitored and information required to continue their care without delay omitted.

The results from our in-depth interviews explored the perspectives of health care workers involved in the care of critically ill patients and demonstrate how the occurrence of these problems affect care in different ways during the Receive, Sustain, and Flow phases. We used **Table 1**, **Table 2** and **Table 3** to illustrate how the data supports the themes we have developed and align with SEIPS categories then explain how the data related to Receive, Sustain, and Flow in the paragraphs below.

**Table 1:** Themes and accompanying quotes reflecting the issues impacting receipt arranged according to SEIPS categories

SEIPS categories	Tools and technology	Person	Organisation	Tasks	Physical environment
Receipt themes 1of 2	Lack of equipment for initial triage.	Inadequate nurses and doctors required to receive patients	Training limited to specific staff groups	Specific staff required to receive patients	Inappropriate bed spaces to receive patients
Quote and affected hospital areas	*“… sometimes there is no BP machine may be the battery has been finished, I mean there are some minor challenges which hinder proper provision of services.”* OPD, Nurse, Facility D, TZ	“*But now you find me there alone, with all those patients. I will not- I will not be able to capture all of them. At least maybe out of 5 children who really need first priority, I’ll manage to consider, to identify 1 or 2, the rest I shall not have captured but they are just in the midst of this big group*” OPD Nurse, Facility E, KE	*“.. For the going on trainings it is really to involve attendants, it is very rare for them to get full package of the training*.” OPD and Wards Nurse, Facility E, TZ	“*so the first person who meets the patient is the triage nurse, a qualified nurse*” OPD, Nurse, Facility D KE	“*you know now the challenge always comes in because you don’t have the bed which you can support the portable machine so someone has to carry the oxygen with the patient, but we have the cylinders*.” Nurse, Facility B, KE
				*“…so when the patient walks in there is a triage area where there is a medical officer and a nurse so I think the patients will be catered for in view of their presenting symptoms so that the one that needs the most urgent care is the one that will be cared for first.”* Doctor, Facility C, KE	
Aspects of work affected by payment		Inconsistent payment waiver systems			
Quote and affected hospital areas		“*Other challenges are our patients, most of the time are not insured, you find a patient absolutely needs electrolytes what, what, what, but he/she simply tells you he/she I have no money, simply that I don’t have money. So as the head of department you need to think beyond, you may try now to go for exemption option but then you may find the patient has already paid something at OPD, you know, there are protocols with financings, I don’t want to know about them, but the challenge is most of the patients cannot afford treatment or investigations.”* Doctor, Facility B, TZ			
Receipt theme 2 of 2		Use of students to overcome HR shortages	Scant protocols	Patient care delayed due to staff shortage	
Quote and affected hospital areas		“*We, we have a nurse who is stationed there at…at the outpatient who actually does triage with conjunction with the, with the, with the, some of our [student] volunteers…some of our volunteers that are…we have at the facility, that help the at facility…occasionally when the nurse is overwhelmed*.” OPD, Doctor, Facility E, KE	“*We would require protocols and all staff need to be trained about these protocols. That if a patient presents with this symptom, what next? You know, people don't just walk around without you know, knowing what to do*.” Wards, Doctor, Facility E, KE	*“Of course, there are sometimes delays because we can have a critically ill patient but is also there is another critically ill patient so you can't leave one and take care of the next one so you have to finish with the one that is already there*.” OPD, Doctor, Facility C, TZ	

SEIPS – Systems Engineering Initiative for Patienet Safety, OPD – outpatient department, KE – Kenya, TZ – Tanzania

**Table 2:** Themes and accompanying quotes reflecting the issues impacting sustentation arranged according to SEIPS categories

SEIPS categories	Tools and technology	Person	Organisation	Tasks	Physical environment
Sustain theme 1/2	Lack of equipment for maintaining patients	Lack of formal routines to catch deterioration	Rationed care	Referral used to manage basic shortages	Layout adaptations not built for purpose; lack of design for deterioration
Quote and affected hospital areas	“*Ok, is there any way of providing oxygen to patients at the OPD before he/she arrives here? No, there is not…if the patient can’t breathe they come to take oxygen or they come and say that there is a patient who need oxygen then I will give them a portable cylinder so that he/she can use it when transferring a patient*.” OPD, Nurse, Facility C, TZ	“*In the wards there is also shortage, so they just check in general and they if they deteriorate there is resuscitation and they make it, sometimes they don’t make it*” Wards, Nurse, Facility B, KE	“*it is never there throughout, like today, it was not there in the whole facility apart from casualty, we had, because we have the piped oxygen*” ED, Doctor, Facility D, KE	“*The only high flow nasal oxygen we have is of course the option of using the non-rebreather masks which will give us fifteen litres per minute. That's the only option, after that if there is no improvement, then you think the patient needs intubation then that is above our level now we'll have to refer.”* Wards, Doctor, Facility B, KE	*“… according to the capacity of that work the way it was designed, each cube was supposed to have only one bed, but we are forced to put two beds. And there is a season where you have so many clients that you find yourself having two beds in each cube. But there have been some broken beds which have not been replaced, so sometimes patients are forced to sleep two in one bed, and in some instances we are forced to put mattresses over the floor, for patients to sleep. The ward overflows sometimes*.” Wards, Nurse, Facility D, TZ
			*“Due to insufficient number of staff, also you might have many patients and you cannot go around them all, but when they are few we normally do that.”* Nurse, Maternity, Facility E, TZ		
Aspects of work affected by payment	Inability to pay limits management options				
Quote and affected hospital areas	“*That’s a bit of a challenge. That's a really, really bit of a challenge. Because sometimes we run out of oxygen, we have to wait for one or two days, you know, yeah, so patients who really urgently require oxygen they either have to buy their own oxygen or we have to refer them. And most of the time they end up dying either on the road or just in the hospital.*” Wards, Nurse, Facility E, KE				
Sustain themes 2/2	Basic treatment inconsistently available	Neglected comms systems	Absent equipment and medication management systems	Subjective assessment escalation decisions are made	Obstructive hospital design on wards
Quote and affected hospital areas	“*they are very sick but can able to talk to you, able to swallow but now they ask you for water for, for taking the medicine, so you don’t have water for taking the medicine. So, we don’t even have somebody to offer us even a dispenser just that, so that patients who are told to take the medicine and can always at least swallow their drugs. So sometimes the relative is to go…to the gate or to XXX (a nearby shopping store), buy a bottle of water and come back even after 30 minutes or 40, and this patient is already with the drug wants to take the drug. So those are some of the challenges”* Wards, Nurse, Facility B, KE	“*Sometimes we have the challenge because it is out of credit, and because it is a big phone that we are using, sometimes it goes out of charge, and then people hesitate to use their charger because it kills the charger. There was a point where its charger was stolen or lost*” OPD and Wards, Nurse, Facility D, TZ	“*the consumption is high as well at casualty and there was no oxygen cylinder in the whole facility, it means it’s not going to be changed even tonight. So maybe in the middle of the night it will get over. If you happen to get a patient who really needs oxygen it will be a big, big challenge, but we live with such challenges*.” ED, Nurse Facility D, KE	“*The MO interns in the wards, they are being called by the nurses when the notice the patient is deteriorating the MO intern has to examine the patient then according to his/her examination they decide to call the consultant the patient needs to be referred, so it is mostly the MO intern who decides*” Wards, Nurse, Facility B, KE	“*So that one we are unable to do…to monitor the patient the patient the way we would like to. Because we can’t see them. We just depend on them to call us or their neighbours or something. Yeah, I don’t know whether…we are in charge of the patients but sometimes we are also handicapped*.” Wards, Nurse, Facility A, KE

SEIPS – Systems Engineering Initiative for Patienet Safety, OPD – outpatient department, KE – Kenya, TZ – Tanzania

**Table 3:** Themes and accompanying quotes reflecting the issues impacting flow arranged according to SEIPS categories

SEIPS categories	Tools and technology	Person	Organisation	Tasks	Physical environment
Flow themes	Lack of equipment delays flow of care	More obstacles when transferring patients not admitted	Risk taking to maintain flow	Absence of plans beyond referral spaces	Space necessary for continued hospital journey
Quote and affected hospital areas	“*Sometimes machines are not working so when you go for investigations you will be told that the machine is not functioning therefore you cannot take him/her to the ward without investigations and results.*” Services, Nurse, Facility E, TZ	“*the driver is…can easily escort the patient for chest x-ray as an inpatient. So, if it is an inpatient, [or] so if it is an outpatient sometimes it’s never easy, it’s never easy.* ” Services and Wards, Nurse Facility E, KE	“*So sometimes those cylinders they run out of oxygen and there can be a time before we are able to settle the patient, that the patient is out of oxygen…which the patient will already have missed by the time now we are ready to deliver oxygen.*” Wards, Clinical Officer, Facility C, KE	“*sometimes you want to refer but you are not able to refer because maybe the facility you are referring to thinks maybe they also don't have a bed to accept the patient or something like that so you have to struggle with the limited resources*. ” Wards, Nurse Facility B, KE	“*they do wait. The place is very busy, so you find so many patients… So maybe you find a patient waiting to be transported to the ward. Yeah, but maybe for like an hour while we are organising for beds*” OPD and Wards, Nurse Facility A, KE
			*“Most of the patients come from the OPD. We expect Dr XXX will do those ABC (Airway Breathing Circulation) and hand to me a patient who is stable, but the challenge is there is no infrastructure so as to stabilise the patient. So we may receive a patient with hypoglycaemia, but we realise it in the ward.….. mostly we receive patients from OPD but sometimes in very unstable condition*” Emergency Department, Nurse, Facility B, TZ		
Aspects of work affected by payment		Payment exceptions made for very sick patients			
Quote and affected hospital areas		“*Yes, and most of the time we get challenges – you find that maybe the ICU at XXX is full…we also have options like private facilities but you find some of the patients that we get they can’t afford the private because they [private hospitals] always ask for a deposit. So those patients end up in our wards, we always do our best in our wards now*.” Wards, Nurse, Facility C, KE			

SEIPS – Systems Engineering Initiative for Patienet Safety, OPD – outpatient department, KE – Kenya, TZ – Tanzania

**Correspondence to:** Jacob McKnight Tropical Medicine Nuffield Department of Clinical Medicine Oxford, England UK jacob.mcknight@ndm.ox.ac.uk

In-depth interviews provided further evidence for the three phases throughout the care of critically ill patients. Quotes illustrating this are laid out in Appendix 3 in the **Online Supplementary Document**.

### Receive phase

We define “Receive” as the phase during which patients who are critically ill, or who are at high risk of becoming critically ill, arrive at a location within a facility until they are officially under definitive care of a facility or department. Receipt processes were based on ad hoc practices that are normalised rather than a specific design. We find they have many weaknesses, with considerable potential for harms as they do not always include formal triage and rely on the discretion of whoever is available to identify critical illness.

The different departments and hospitals we visited had almost always developed a de facto “system” for receiving patients. In some situations, this amounted to semi-formal triage that considered severity and need (**Table 1**).

Receipt processes also happen in departments within the facility, and issues of readiness to receive patients and failures regarding the status of patients being received were common. Additionally, some spaces were more associated with critical illness than others (**Table 1**).

Nurses with a specific mandate to assess arriving patients often worked in settings with insufficient staffing which meant patients were not officially admitted, and sometimes missed timely identification or care. Even where the local system was designed to work well it was undermined by an array of factors (**Table 1**).

As shown in **Table 1**, **Table 2** and **Table 3** receipt processes were considerably hindered by payment problems and by bed space. Payment problems were unique in affecting multiple SEIPS categories simultaneously because if patients could not pay, this would delay their admission, receipt of treatment while waiting for admission (**Table 1**). In **Table 1**, when a patient presented at the receipt phase, inability to pay affected the person, organisation and task because staff present describe being unable to provide treatment deemed necessary for patients arriving into their care and the hospital system was unable to provide the necessary.

### Sustain phase

We define “Sustain” as the phase during which patients who are critically ill or who have a high risk of becoming critically ill are formally assessed and receive interventions to keep them stable under the care of a specific team. Unfortunately, even when a patient is under the care of a hospital team with or without a management plan, their issues can go unseen before or after critical illness is identified as illustrated in **Figure 2** and Appendix 3 in the **Online Supplementary Document**. We consider a hospital team to be a group of people working under a specific clinical remit that may or may not be confined to a specific physical space within the hospital. For example, fundamental issues with staffing meant that patients who were becoming critical might not be identified (**Table 2**).

The lack of formal management systems for managing the treatment of critically ill patients was evidenced by the presence of informal normative systems i.e. for communications, where staff used mobile phones when hospital phones were non-functional. We also noted that payment issues interrupted care during all phases, transcending multiple SEIPS categories (**Table 2**). Those who could pay continued to receive care while some who could not, had their treatment halted or were referred to other facilities to manage their case.

Patients who cannot advocate for themselves due to their physical condition suffer the most when poor sustentation systems persist within a facility.

### Flow phase

We define “Flow” as the phase during which patients who are critically ill or who risk becoming critically ill are waiting for external services or are in transit between different locations in a facility

Flow issues can arise when patients cannot be moved to departments and end up stuck in limbo (**Table 3**). Flow problems also emerge when patients are not adequately stabilised before being moved to departments outside of OPD or the emergency department.

This also happens with laboratory and radiology tests as there were often no formal priority system in any hospital visited. The absence of a structured approach to managing critically ill patients during the flow phase limits provision of a consistent quality of care, especially EECC.

## DISCUSSION

Receive, Sustain, and Flow are generic, recursive phases undertaken by all health facilities during hospital journeys and illustrate the multiple transitions a patient experiences in any one patient stay. It is our contention that any patient who is or who could become critically ill can be considered to be in one of these three phases from their arrival in the medical facility until they are discharged (**Figure 2** and Appendix 3 in the **Online Supplementary Document**), and can also be witnessed in similar journey analyses conducted elsewhere (Appendix 4 in the **Online Supplementary Document**).

We believe RSF is valuable because it is a simple heuristic that may not require detailed investigation but allows teams with limited resources to reflect on the areas and processes within their hospital that negatively impact the care of critically ill patients. There are many tools available to those who wish to improve patient safety [8,24-26]. Quality improvement approaches have been used extensively in African hospitals as they have in hospitals around the world [27,28]. Process mapping and improvements based on measurement and redesign remain powerful tools for reformers, but in settings such as those described here and possibly those in higher income settings, hospitals may lack the resources required to conduct detailed, granular analysis of root cause issues, careful measurement and redesign [29-31]. Similarly, while quality improvement approaches may be highly appropriate for well-defined areas of hospital areas, it would be a significant undertaking to try to use these methods to identify the root cause all problems affecting the care of critically ill patients across their hospital journeys whereas our heuristic offers a more simplified approach to uncovering problems affecting the care of critically ill patients and potentially other patient groups.

Tools such as SEIPS offer practical, universal approaches to safety that are likely to be effective for African hospitals but they lack the narrative, temporal power of hospital journeys [30,32]. Systems Engineering Initiative for Patient Safety 3 focuses very much on patients and has moved from the linear approach of Donabedian to a more cyclical approach that shows both a diagnostic cycle encapsulated in the work system and as outcomes feeding back into the work system [24]. Systems Engineering Initiative for Patient Safety is not temporal however and while it increasingly encourages a patient-centric view, it cannot reveal the personal details associated with the hospital journeys described above. The same can be said for the WHO Emergency Care system framework which focuses on opportunities for improvement between the pre-hospital and emergency unit phase of a health emergency but offers minimal insight into how to address the care of critically ill patients once they are within the hospital system, beyond the emergency department [33]. Likewise, technology and training have been unable to produce the sustained results required for long term quality improvement as they do not address many of the temporal administrative problems that plague many facilities [34]. We anticipate that addressing these problems, such as having someone take charge of maintenance for equipment, communication tools etc. is likely to improve the implementation of EECC, as these issues are not directly EECC related but could potentially limit the benefits of EECC for critically ill patients.

It is important to recognise however, that although we believe all hospital journeys within facilities can be analysed with the RSF heuristic, we are not attempting to replace quality improvement, process mapping, nor patient narrative analyses. Instead, we seek to provide a language to describe issues that arise in the identification and treatment of critically ill patients for busy health facility managers who do not have access to resources for more involved improvement efforts [4]. The need for easier to use, simpler models for applying patient safety thinking was recently raised in a review of SEIPS studies and in response, Holden and Carayon created “SEIPS 101” that includes a “hospital journey narrative” approach [35,36]. The approach outlined here could be considered as a further variant of the types of journey maps provided but with a patient-focused, critical care emphasis.

This is particularly the case as we saw no need to iterate or move beyond the categories of issues established as part of the SEIPS framework [37]. Our initial inductive findings, captured in Gioia tables (Appendix 5 in the **Online Supplementary Document**), were remapped onto SEIPS categories with little loss of clarity. There is one area, however, that seems to lack emphasis within SEIPS, but was central in our findings namely patients’ ability to pay for facility-based health care services. The fact that (in)ability to pay constrains access to critical care in Africa has been recognised but solutions have clearly not been realised in Kenya nor Tanzania [38,39]. This was a critical issue in all three phases and slowed and confused clinical decision-making. Therefore, while our tool (Appendix 5 in the **Online Supplementary Document**) uses the SEIPS categories for analysis of each phase in each department, we have also added a new “Payment” category (External influence) to ensure that issues pertaining to patients’ access to care are properly captured. We have also set out simple guidance in Appendix 6 in the **Online Supplementary Document** that we believe could guide the process of mapping of hospital journeys using the RSF tool. Together, we hope that they can be used by health facility management to apprehend the full gamut of issues that affect the care of critically ill patients across their whole journey through the health facility.

### Limitations

We anticipate that changing the mindset of people working within the hospitals we have discussed in this paper from one of managing on a shift-by-shift basis to one where they understand their role in a complex system will limit the implementation of RSF. The immediate demands of working life in the hospitals we studied is a significant limitation on improvement efforts of any kind. While RSF should not require significant financing, it still requires support from senior managers and this may be difficult to attain, especially where the conclusions of its use suggest investment or reallocation of resources. The extent to which the simplicity of RSF offers advantages in implementation over other quality and safety approaches has not yet been established. Further trialling and testing of this approach should reveal the extent to which management, political, or financial support are required to make the most of the RSF approach and should also offer further opportunities for optimisation and improvement of the tools.

## CONCLUSION

Receive, Sustain, and Flow provides a simple framework for care improvement by addressing aspects of work that are not directly clinical but do have an impact on clinical work at a health facility. Its simplicity makes it accessible for those with limited time and resources to dedicate to quality improvement endeavors while allowing them to meaningfully identify their own areas for intervention without the need for expert quality improvement teams. We believe that when trialled in practice, RSF would allow those working at facilities to look at problems from either a whole hospital point of view or that of individual connected departments depending on the time and staff available to partake in using it. Additionally, RSF could help hospital and ward managers advocate for technology, staff or equipment that the RSF tool demonstrates could improve the chances of survival for critically ill patient. Furthermore, this data generated the RSF tool could help hospital teams re-evaluate the allocation of staff and other resources that have an impact on the survival of critically ill patients. Finally, RSF could provide a uniform language that can be used across departments to communicate identified areas for change and potentially increase the likelihood of cross departmental improvement efforts, especially when improving care of critically ill patients.

## Additional material


Online Supplementary Document
