# Supplementary material for: Receive, Sustain, and Flow: A simple heuristic for facilitating the identification and treatment of critically ill patients during their hospital journeys
Source: J Glob Health. 2023 Dec 22;13:04139. doi: 10.7189/jogh.13.04139 (PMC10740342; doi:10.7189/jogh.13.04139)
Supplement: Online Supplementary Document [file jogh-13-04139-s001.pdf]

Appendices

Appendix 1. SEIPS3 illustrative diagram. Original image taken from Carayon et al 2020. (P. Carayon et al., 2020)

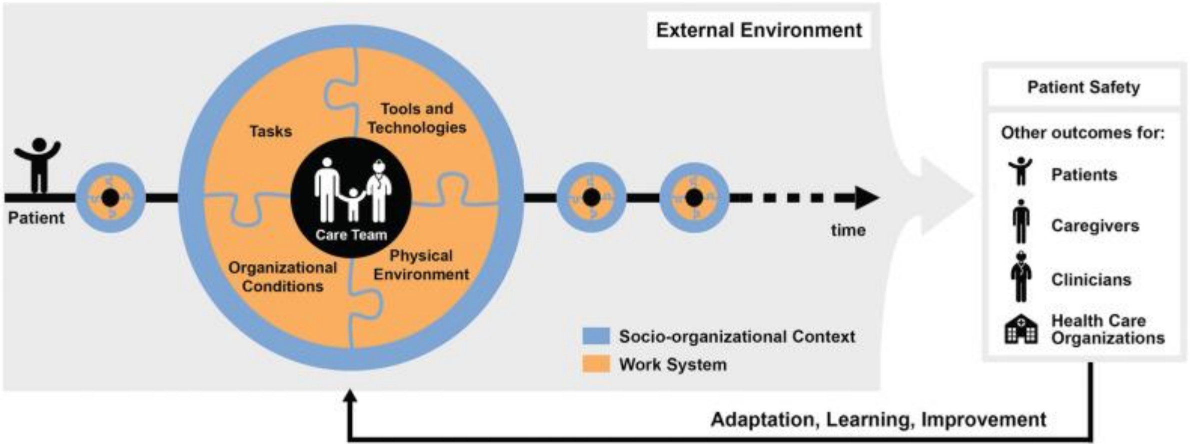

Appendix 2. Demographic details

Table 2.1 Participant demographics table

	Kenya	Tanzania
**Gender**		
**Female**	10	9
**Male**	6	6
**Interview mode**		
**In-person**	0	15
**MS Teams**	1	0
**Phone**	15	0
**Cadres**		
**Clinical Officer**	11	0
**Medical Officer**	3	6
**Nurse**	2	9
**Departments**		

**A&E**	6	4
**ICU**	1	2
**Internal Medicine**	1	3
**Isolation Unit**	1	0
**Management**	3	0
**Maternity**	2	2
**Outpatients**	2*	3
**Paediatrics**	1	1
**Surgery**	0	1**
**Unlisted**	1	0

***OPD combined with A&E, ** Surgery role combined with Internal Medicine**

Appendix 3 RSF evidence from results

3.1 Hospital Journeys

Box 2. Facility B Kenya: A hospital journey of a 29-day old baby in the new-born unit

Receive Sustain Flow

At 10:00 Baby X is on a resuscitaire receiving a blood transfusion (transfusion began before our observation). Baby X has spent 29 days in the newborn unit diagnosed with prematurity respiratory distress syndrome and anaemia. This baby is receiving incubator care, nasogastric tube feeding and oxygen via nasal prongs. This baby is admitted within a unit that has 46 babies split between two acute rooms. Term babies ins room A (11 babies) and preterm babies in room B (18 babies). The other 17 babies are under incubator care receiving either nasogastric feeding or oral feeding. Room A and room B are managed by one nurse each per shift and four student nurses between them. One medical officer and one study nurse (not part of the nursing rota) also provide medical care within the unit. Baby X is also receiving oxygen via nasal prongs. The Medical Officer intern is conducting a ward round.

At 11:05 a student nurse comes to monitor progress of the transfusion. The student takes the baby's vital signs (temperature, respirations, heart rate and SPO_2) and documents them in the file. Thirty minutes later, the transfusion is concluded. The student nurse takes vitals once more and documents. Baby is moved back into the incubator. At 12:05h the mother comes to feed the baby. She expresses the prescribed volume of breastmilk (EBM) and feeds the baby via the nasogastric tube (NGT). The nurse is attending to an admission at the admission bay. An hour later, the nurses hand over to the afternoon staff. Two nurses have reported for the afternoon shift – one for each Acute room. Two more babies are waiting in the admission bay. At 13:47 two new student nurses have reported for the afternoon shift. The students are updating the nursing cardexes. One more baby has been admitted to this acute room (total of 18 now).

Ten minutes later, the Medical Officer intern completes the ward round and leaves the unit. At 14:20h a student conducts a random blood sugar (RBS) check for the babies in Acute room B. RBS for Baby X is 5.9 mmol/L. Forty minutes later, Baby X's mother comes for the 3-hourly feeding and notices a change in the baby's breathing. Mother communicates her concern to the nurse who assures that the distress will resolve soon. Mother reminds the nurse that the NGT needed to be changed in the afternoon. The nurse changes the NGT and mother feeds the baby on expressed breastmilk via NGT. A student nurse conducts an oxygen saturation check, noted to be 97%. There's one more admission into the other acute room.

At 18:13 mother comes to feed the baby. Expresses the prescribed volume and feeds the baby via NGT. Fifteen minutes later one nurse reports for the night duty. The other nurses hand over to her. One more nurse is expected to come (2 nurses in the nightshift).

This hospital journey focuses mostly on the treatment of a baby during the Sustain phase. However, possible deterioration still occurs although promptly noticed by the mother and acted upon by staff. As observations of this hospital journey began after admission, observed periods of receipt are only noted when the patient's care is transferred to members of staff during shift changes and for new admissions which do not affect the staff's ability to care for the critically ill patient. One moment of flow occurs where the patient is moved within the ward but is not monitored during or immediately after this move.

Box 3. Facility D Kenya: Journey of a patient who presented with symptoms of severe difficulty in breathing (Covid-19 patient).

Yellow	Receive
Green	Sustain
Blue	Flow

At 0830 a patient is wheeled into the casualty [ER] with apparent symptoms of severe breathing difficulties. The patient is being wheeled onto a couch in the casualty. The Emergency Room (ER) and the general outpatient are housed under one roof. The unit has one nurse and one Medical Officer and is full of patients waiting to be seen. The Medical Officer is still not yet in. The doctor [Medical Officer] was here overnight, so he has stepped out to refresh before coming back. The medical officer is also covering theatre in case of any surgical emergencies. The nurse reported at 7:30am and will oversee this unit until 12:30pm when another nurse will come. Upon coming back we hope the patient will be reviewed and if at all there are any investigations to be taken the patient will be taken accordingly. The other staff in the department include a few nursing students assisting in the care of these patients. There are also two Clinical Officers; one here in the emergency room, the other one covering the outpatient section.

Five minutes later, the vitals are taken. It has been noted that the heart rate is a little unstable, ranging as low as from 50s to 60s but at times it's fluctuating so not really stable. She's having breathing difficulty; the pulse oximetry reading is 58% when the patient isn't receiving additional oxygen. The unit has only one pulse oximeter that is operational. There is another one attached to the monitor but not working. The other patients…there is one other patient who needs constant pulse oximetry, but he has his own…the family came with a personal pulse oximeter, so I think for that he's sorted for now. The ER has three [3] beds/couches for patients, and at the moment, all three beds are occupied

Thirty minutes after the patient first arrived, the doctor arrived. He has reviewed the patient as well as the others. He's suggesting an X-ray, so the relatives have gone to the registration window to open a file for the patient. So, we await that after which the prescribed test [X-ray] …she'll be wheeled in.

0915h: The Patient has just been wheeled in for an X-ray. Being that she is among the very first patients who reported to this casualty we are hoping that it won't take long and she's going to be attended to almost immediately. So, we hope that she'll be out in the next few minutes. Twenty minutes later the patient leaves the X-ray department, awaiting review with results [results are not out yet] …so, the patient is back in the casualty. A further twenty minutes later, the patient has been put on oxygen, in bed at the casualty department awaiting the X-ray results. Not really connected to a monitor because the monitors are faulty, but the patient is on oxygen, so from time to time the vitals are being checked. The unit has one functional port for piped oxygen which is currently in use. the place is full as at now. There is an oxygen cylinder in the ER, but that as well is in use by another patient.

Twenty five minutes later, the X-ray results are out but there has to be a Covid test done. The Covid rapid test is not available in that department so the patient has to be taken elsewhere in the hospital for the test. The relatives are still following with whoever is responsible for how best that can be done, but otherwise the patient is still on oxygen, we are awaiting review with the X-ray results. There is a queue…there's one patient ahead so the Medical Officer is still with another patient but once he's done, I think the patient will be reviewed with the X-ray results. However, we are still following up with the Covid team for the Covid test. I'll keep you posted.

At 10:43 the Covid team came to take samples for the rapid test from the casualty. The samples are being taken and the results will it'll inform whether this patient is admitted in the medical ward or the Covid isolation ward. At **11:27h** the test results for Covid-19 are out, she is confirmed to be Covid positive. So, the patient will be admitted in the Covid isolation ward. Initially they had planned to admit her in the female medical ward, but now that the results are out, she's going to be taken to the isolation unit. It has a total of 6 beds. At the moment four of those are occupied, so we are hoping that this patient is going to get space. We'll continue waiting in the casualty. The patient is still on oxygen via face mask, the vitals-the SPO2 is okay. She's saturating on 97% on oxygen. Her pulse was low at 52, at one point even at 43 beats per minute. She's still on oxygen, pulse rising to above 68.

1140h: The vitals have been taken again. The SPO2 is ranging between 90-93% on oxygen. To be noted again is that there is no high-flow oxygen in this facility due to the oxygen supply [strained]. Again, in the casualty there is an oxygen port which is being used to give oxygen to this patient via mask. There aren't any non-rebreather masks in the department as at now but hopefully the saturations will rise. This unit is actually very full and there is only one medical officer. Also there's another patient who is having similar issues [as Patient 2] and they [the medical team] were trying to locate a bed space for the patient…there has been a push-and-pull between the Medical Officer here and the destination hospital [JOOTRH], they have been arguing over phone and the Medical Officer seems frustrated here. Looks like he feels his hands are tied and the patients need help and they cannot be able to help but we are hoping that things ease up over the next few minutes.

1254h: It has been confirmed that there is a bed space for this patient in the Covid isolation ward at this hospital. Her latest SP02 was 94%. So, we are waiting for a stretcher to be brought so that the patient can be wheeled there. Ten minutes later the patient is put on the stretcher to be taken to the Covid isolation ward…the patient will be admitted in the Covid isolation ward. Patient is to be taken there on portable oxygen in a cylinder. They're setting that up.

1319h: Update for Patient 2, the time is 1319h. The patient has just been transferred to the Covid isolation unit and admitted there. To be noted is that this patient is currently saturating at 94% on oxygen but on admission [when she landed at the casualty] the patient's saturations were lower than that off oxygen.

This hospital journey demonstrates that even for patients who are critically unwell, multiple delays can occur during the receipt, sustention and flow of patients. In this case, the patient was only assessed by a doctor and has investigations requested 30 minutes after they were received into the department. Additionally, delays in sustention occurred due to limitations in flow owing to friction in the referral process during which clinical signs fluctuated and they remained in a critical state.

3.2 RSF evidence from IDIs

	Receive	Sustain	Flow
Department	*ED*	Maternity(ward)	ED to Labs(services)

Within facility	*"In triage, when we talk about triaging here we have three categories, the first category is for all patients with emergency cases, the second one is for priority cases and there is a category for key patients"* **Nurse, Facility A ,TZ**	*"Due to insufficient number of staff, also you might have many patients and you cannot go around them all, but when they are few we normally do that."* **Nurse, Facility E, TZ**	*"Sometimes machines are not working so when you go for investigations you will be told that the machine is not functioning therefore you cannot take him/her to the ward without investigations and results"* **Nurse, Facility E, TZ**
Department	Obstetrics and Gynaecology	Infectious Diseases	Facility to Facility
Within facility	*"…so when the patient walks in there is a triage area where there is a medical officer and a nurse so I think the patients will be catered for in view of their presenting symptoms so that the one that needs the most urgent care is the one that will be cared for first."* **Doctor, Facility C, KE**	*"Yes, there are nurses in the wards who keep checking on these patients, let's say a patient is in DKA this patient has to be monitored, the sugars…every one to two hours. So there are nurses who monitor the patient assisted by the clinical officer or MOs in the ward."* **Doctor, Facility B, KE**	*"…we need more of the guidelines. The SOPs are very necessary: the triage and the referral systems. You can imagine the referral system, a patient goes…comes from Kihara, Kihara is next to Nairobi, the patient has to come to Kiambu, then is referred to Kenyatta [KNH]. You want to refer the patient to Kenyatta – we do not have a CT scan. The patient goes to Kenyatta for the CT Scan, then comes back to be reviewed with the CT Scan, then referred again to Kenyatta [KNH]. Will that patient survive?"* **Nurse Manager, Facility B, KE**
Department	ICU	ICU	ED to ward
Within ward/department	*"It happens sometimes, the confusion happens between the doctors and nurses. This happens for instance I receive a patient from general ward with full*	*"Triaging means sorting, it means to sort the patients in terms of severity and which patient should be given priority. This helps to serve the patient on time, this means early recognition early treatment. So there*	*"Most of the patients come from the OPD. The challenge is the infrastructure at OPD I hope you have seen it, is quite different from emergency set up. We expect Dr XXX will do*

	consciousness and awareness; this a patient will need supportive management at that time without a doctor. So when a doctor comes and ask me on which criteria did I receive the patient. So sometimes we lack more information. So that happens" **Nurse, Facility A, TZ**	*are some weaknesses in general wards in terms of sorting. This is caused by poor sorting meaning late recognition of a patient as the patients become many compared to number of nurses and doctors. This affects triaging."* **Nurse, Facility A, TZ**	*those ABC and hand to me a patient who is stable, but the challenge is there is no infrastructure so as to stabilise the patient. So ,we may receive a patient with hypoglycemia but we realise it in the ward. A patient may be brought to you and you are told that the patient is too ill, rush him/her to the ward, the patient needs oxygen, rush him/her to the ward, and the oxygen is not available there…..in mostly we receive patients from OPD but sometimes in very unstable condition*" **Nurse, Facility B, TZ**
Department	ED	ED	ED
Within ward/department	"*So critically ill patients when they come, we classify. Nowadays we've started to classify… o we are using the South Africa triage system whereby we have at least started coding the patients. We are using the red, orange, green and yellow… we fill everything and any patient who appears to be red there is…we tag the patient then we take him or her to the emergency area. And we ensure that the patient is attended to immediately and is taken where the, the definitive care,*" **Doctor, Facility D, KE**	"*I think for the nursing part what we do at least we take vital signs four hourly. And you also reassess the patient every time using the GCS, it can also assist.*" **Nurse, Facility D, KE**	"*…we have, right now we have CT [CT scan], we have chest x-ray, I think we have almost all, MRI, we have all the radiological examinations being done. But now the problem here is, that is where the problem comes; Money. Yeah, money. You see, like a CT is 5000 [Ksh], and a patient who has come with trauma, you cannot waive 5000 [Ksh]. That is where now the problems come… Maybe this patient has come, he does not have money. So it takes a bit, a bit longer for the patient to be done the radiological [tests] because of the finances, because you have to pay cash*" **Nurse, Facility D, KE**

Appendix 4. Patient journeys from the literature

Box 4. Patient journey reconstruction (Benson et al., 2022)

Reproduction of a patient journey extracted from Benson 2022 et al

"I don't know what is happening to my body and my husband and I are both anxious. I feel as if I am losing my identity and my spontaneity and I'm afraid that my husband will stop loving me." She often uses her hand to keep her head still When her stress level calms down a bit, the symptoms unfortunately do not disappear but intensify so that she can no longer go jogging and riding and has been on sick leave for a few weeks.

Multiple visits to her GP who prescribes strong pain killers and muscle relaxants and refers her to different specialists. Most frequently suspected diagnoses: stress, psychological causes, herniated disc, tendonitis.

Lilly's symptoms have worsened, and she cannot keep her head still and straight. Her sleep quality is very poor, she is depressed and suffers from fatigue. Her GP finally refers her to a neurologist specialised in dystonia who diagnoses her with "CD". She does not receive clear information about the disease or support from the diagnosing neurologist. In an online article, she learns more about the diseases and its treatment.

Lilly's neurologist refers her to a movement disorder clinic where it is recommended sheget BoNT injections. The neurologist also suggests physiotherapy in addition to the injections but doesn't direct her to a physiotherapist who is knowledgeable about dystonia. He advises her to search for a physiotherapist in her area, one that ideally specialises in CD.

She often struggles with fatigue which is caused both by the painkillers and by poor sleep quality. She hopes that the pain will get better with time.

Box 5. Extracts from a patient journey map: Experiences and needs of patients with (suspected) INOCA. CAD=coronary artery disease; CFT=Coronary function testing; CVD= Coronary Vascular dysfunction; GP= general practitioner; HCP=Healthcare Provider; SDM= shared decision-making

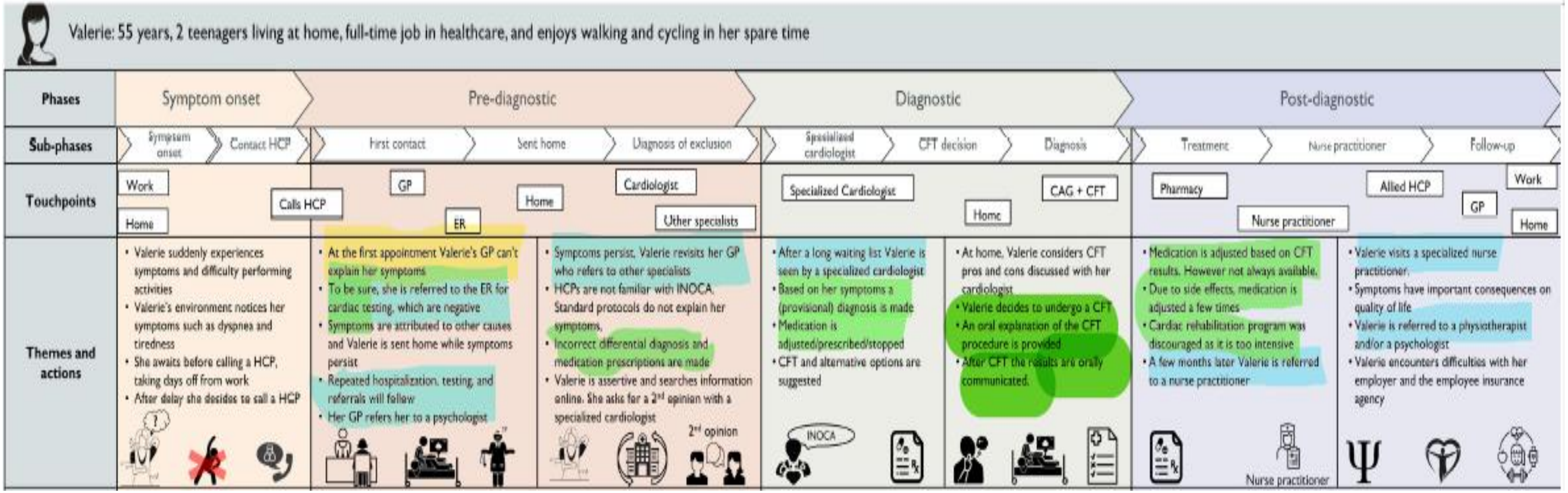
Valerie: 55 years, 2 teenagers living at home, full-time job in healthcare, and enjoys walking and cycling in her spare time
Phases
Symptom onset
Pre-diagnostic
Diagnostic
Post-diagnostic
Sub-phases
Symptom onset
Contact HCP
First contact
Sent home
Diagnosis of exclusion
Specialized cardiologist
CFT decision
Diagnosis
Treatment
Nurse practitioner
Follow-up
Touchpoints
Work
Home
Calls HCP
GP
ER
Home
Cardiologist
Other specialists
Specialized Cardiologist
Home
CAG + CFT
Pharmacy
Nurse practitioner
Allied HCP
GP
Work
Home
Themes and actions
• Valerie suddenly experiences symptoms and difficulty performing activities
• Valerie's environment notices her symptoms such as dyspnea and tiredness
• She awaits before calling a HCP, taking days off from work
• After delay she decides to call a HCP
• At the first appointment Valerie's GP can't explain her symptoms
• To be sure, she is referred to the ER for cardiac testing, which are negative
• Symptoms are attributed to other causes and Valerie is sent home while symptoms persist
• Repeated hospitalization, testing, and referrals will follow
• Her GP refers her to a psychologist
• Symptoms persist, Valerie revisits her GP who refers to other specialists
• HCPs are not familiar with INOCA. Standard protocols do not explain her symptoms.
• Incorrect differential diagnosis and medication prescriptions are made
• Valerie is assertive and searches information online. She asks for a 2nd opinion with a specialized cardiologist
2nd opinion
• After a long waiting list Valerie is seen by a specialized cardiologist
• Based on her symptoms a (provisional) diagnosis is made
• Medication is adjusted/prescribed/stopped
• CFT and alternative options are suggested
INOCA
• At home, Valerie considers CFT pros and cons discussed with her cardiologist
• Valerie decides to undergo a CFT
• An oral explanation of the CFT procedure is provided
• After CFT the results are orally communicated.
• Medication is adjusted based on CFT results. However not always available.
• Due to side effects, medication is adjusted a few times
• Cardiac rehabilitation program was discouraged as it is too intensive
• A few months later Valerie is referred to a nurse practitioner
Nurse practitioner
• Valerie visits a specialized nurse practitioner.
• Symptoms have important consequences on quality of life
• Valerie is referred to a physiotherapist and/or a psychologist
• Valerie encounters difficulties with her employer and the employee insurance agency

Appendix 5 Gioia Tables

5.1 Receive Gioia Table

First Order	Second Order	Aggregate dimensions	SEIPS
Presence and sufficiency of equipment a consideration when receiving patients	Equipment sufficiency	Failing initial engagement	Tools and tech
Appropriate equipment			Tools and tech
Emergency case training restricted to single department	Training distribution		Org of work
Absence of protocols affect decision making	Scant protocols		Org of work
Registration limited by ability to pay	Financial limitations		
Assistance from medics required when receiving patients (formal requirement)	Teamwork focused on the critically ill	Perpetually constrained emergent systems	Task factors
Nurses and medics required when receiving patients(informal choice, adequate assitance)			Person factors(care team)
Teamwork throughout patient journey			Person factors(care team)
Adapting to capacity constraints using volunteers	Responses to staffing constraints		Org of work
Delaying patient care due to staff shortage			Task factor/personal factor(care team)
Inadequate staff equals less identification			Org of work
Delayed care initiation due to insufficient staffing			Task factor/personal factor(care team)
Constrained care at night			Org of work
Reduced efficacy of triage			Task factor
Unmet patient demand			Org of work
Initiating care without specialists	Impact of competence	When things run smoothly	Org of work
Methodical receipt process			Task factor
Fewer delays			Task factor
Recognition of the needs of critically ill patients			
Equipment sufficiency	Perception of service prerequisites		tools and tech
Specialised training			Org of work
Flexible registration procedure	Making exceptions		External influence
Prioritising space			Physical environment
Payment exception for critically ill			External influence
Available space determine who can be received on wards	Designated spaces		Physical environment
Allocated space guarantees access to care			Physical environment
Space leads to receipt at next stage			Physical environment

5.2 Sustain Gioia Table

First Order	Second Order	Aggregate dimensions	SEIPS
Basic treatment not guaranteed	**Treatment sufficiency not guaranteed**	**Increased chances of care going wrong (holely cheese)**	Tools and technology
Insufficient delivery equipment			
Oxygen prioritised to specific locations	**Distribution of essential treatment**		Tools and technology
Long standing equipment and meds inadequacies	**Essentials and equipment inadequecies**		Tools and technology
Rationing care			Tasks
Increased chances of care going wrong			Tasks +Tools and
Present but insufficient			Tools and technology
Oxygen management incongruent with facility demand	**Disjointed management processes**		Organisation of work
Absent medication and equipment management system			Organisation of work/ tools and tech
Unaligned intra-departmental comms systems			Organisation of work/ tools and tech
Neglected comms system + ineffective alternative			Organisation of work
Inability to pay results in delays	**nflexible payment options**		External influence/ Person factor(patient/client,others)
Inability to pay limits management			
Inability to pay leads to deaths			
Oxygen management limits local escalation option	**Limited escalation options**		Task factors
Referral is sole escalation plan			Person factors(care
Referral used to address basic treatment shortage			Person factors(care team)
Referral is a part of care plans			Person factors(care
Subjective assessment determines escalation time	**Inconsistent escalation strategy**		Person factors/task factors
Mismatched staff to patient ratios	**Inadequate staffing**		Organisation of work
Obstructive hospital design	**Infrastructural layout**		Physical environment
Augmenting activities to suit staff	**Compromised monitoring**	**Compromised HR foundations**	Person factors
Inadequate staff leads to suboptimal monitoring			task factors/person factor
Overwhelmed due to limited capacity			Person factors
Exhausted staff limit management			Person factors
Inadequate staff at night management limits capacity	**Differential care during night shifts**		Organisation of work factor
Patient relatives utilised for monitoring	**Improvisation**	**Making do**	Person factor (others)
Untrained staff utilised for monitoring			factor/organisation of work factor
Relatives used to navigate medication inadequacies			Person factor (others)
Perseverance in unfavourable work environments			Person factor
Oxygen prioritised to specific locations	**Rationing**		Tools and technology
Responsibility placed on nurses	**Allocating tasks**		Organisation of work
Staff provision made for night shift	**Planning**		Organisation of work
Presence of oxygen management			Tools and tech/ org of
Accounting for de-escalation			Org of work
Pre-planned escalation roles			Org of work/person factor(care team)
Standard approach utilised	**Standardisation**		Task factors
Interpretation of vitals readings and standardisation employed			Task factors
Nurses prioritise systematic			Person factors
Categorisation determine next destination for patients and urgency of care	**Care apportioning strategies**		Task factors
Bed availability must be communicated	**Clear communication**		Org of work
Documentation, staff and equipment			Tools and tech
Responsive lines of communication reassuring for nurses- allows them to focus			Tools and tech/ org of work

5.3 Flow Gioia Table

First Order	Second Order	Aggregate dimensions	SEIPS
Care delays due to low staff critical patient ratio	**Delayed Care**	**Consequences of flow delays**	Org of work
When investigations delayed, care is halted and delayed	**Shortage based referral**		Org of work/ task factors
Flow superior to size of space in maintaining care delivery	**Low flow equals increased pressur**		Org of work/Physical environment factor
**Pushing back against patient**	**Ineffective referral- high friction**		Task factor
**Absence of plans beyond referral**			
Inadequate bed space leads to compromise to allow patients to continue care journey	**Flow prioritisation demands rationi**		Physical environment
Delayed care activities	**Level, speed and quality determined by ability to pay**		Task factor/ external environment
Alternative plans made for patients unable to pay			External environment/Org of work
Payment exemptions absent for basic			External environment
Facilitating movement of patients between wards	**Staff presence and adaptation**	**How flow is maintained**	Physical environment
Availability of staff important aspect of effective comms			Personal factor(care team)
Transferring information			Task factor
Space necessary for continued patient journey			Physical environment
Avoidance of patient deterioration through standby nurse presence			Org of work
Maintain patient movement			Org of work/ Personal factors
All staff adapt to different working areas			Personal factors
Specialist staff training essential for managing deterioration	**Specialised training**		Org of work
More obstacles when transferring patients not admitted	**Established admission status**		Org of work
Unaligned services limits work of nurses	**Aligned healthcare services**		Org of work
Calculated risks taken to maintain flow	**Risk Taking**		Personal factor/ Task factors
Space necessary for continued patient journey	**Timely preparation**		Physical environment
Everything must be set in place before transfers			Task factors/Org of work
Availability of oxygen aids in ward			Tools and tech
Improvising to maintain care	**Improvising**		Personal factors
Alternative plans made for patients unable to pay			External factors
Alternative plans made for patients unable to pay	**Methodical communication key**		External factors
Functional line of communication part of continuity of care prep			Org of work/ tools and tech
Back-up care plans for referrals made	**Backup plans**		Personal factors(care team)
Absence of plans beyond referral spaces			Personal factors(care team)
Staff plan for patients unable to pay	**Making exceptions**		External environment
Exceptions made for very sick patients			External environment

Appendix 6. RSF Tool

RECEIVE			
RECEIVE- the phase during which patients who are critically ill, or who are at high risk becoming critically ill, arrive at a location within a facility until they are officially under medical care of a facility or department.	Urgency for facility		
**Tech and Tools**			
Do you have the medical equipment and technical resources you need to be able to receive patients? Things to consider: vital signs monitoring, sufficient beds, space, consumibles (gloves, syringes etc.)	Low	Med	High
**Organisation of Work**	Low	Med	High
Are there systems for checking the status of patients upon receipt? Do you know how to escalate patients who are critically ill? Do you know who to call and will they answer?			
**Tasks**	Low	Med	High
Do you have specific staff assigned to receive critically ill patients? Have staff on this ward received training in identifying and receiving critically ill patients? Does this ward have routines or protocols for receiving critically ill patients?			
**Physical environment**	Low	Med	High
Do you have dedicated spaces for receiving patients on this ward? Is this space always available? Is it sometimes used for other tasks? Do you have sufficient numbers of these spaces? Are there enough beds or chairs in areas where patients are received?			
**External Influence**	Low	Med	High
Are critically ill patients offered receipt services regardless of their financial position? Is there a policy in place outlining this? Are provisions in place to allow departments to comply with the policy? Do they work? Do any other groups outside the hospital play a role in receiving critically ill patients?			

SUSTAIN

SUSTAIN- the phase during which patients who are critically ill or who risk becoming critically ill once a patient has been formally assessed and receive interventions to keep them stable under the care of a specific team	Urgency for facility		
**Tech and Tools**			
Do you have the medical equipment and technical resources you need to be able to sustain patients? Things to consider: vital signs monitoring, sufficient beds, space, consumibles (gloves, syringes etc.)	Low	Med	High
**Organisation of Work**	Low	Med	High
Are there systems for checking the status of patients while they are being sustained? Do you know how to escalate patients who are critically ill? Do you know who to call and will they answer? Is there an established way to communicate when sustaining critically ill patients?			
**Tasks**	Low	Med	High
Do you have specific staff assigned to sustain critically ill patients? Have staff on this ward received training in identifying and sustaining critically ill patients? Does this ward have routines or protocols for sustaining critically ill patients? Is there a system in place for managing the tools required to sustain critically ill patients?			
**Person**	Low	Med	High
Have staff in this ward been trained to sustain staff? Do you have the required number of staff trained to do this during every shift?			
**Physical environment**	Low	Med	High
Do you have dedicated spaces for sustaining patients on this ward? Is this space always available? Is it sometimes used for other tasks? Do you have sufficient numbers of these spaces? Are there enough beds or chairs in areas where patients are received?			
**External Influence**	Low	Med	High
Are critically ill patients offered sustain services regardless of their financial position? Is there a policy in place outlining this? Are provisions in place to allow departments to comply with the policy? Do they work? Do any other groups outside the hospital play a role in sustaining critically			

FLOW

FLOW			
FLOW- the phase during which patients who are critically ill or who risk becoming critically ill are required to wait for external services or are in transit between different locations in a facility.	Urgency for facility		
**Tech and Tools**			
Do you have the medical equipment and technical resources you need to be able to move patients between departments? Things to consider: beds with wheels(trolleys), wheelchairs, portable monitors etc...	Low	Med	High
**Organisation of Work**	Low	Med	High
Are there systems for checking the status of patients while they are being moved bewteen departments? Do you know how to escalate patients who are critically ill while they are being moved? Do you know who to call and will they answer?			
**Tasks**	Low	Med	High
Do you have specific staff assigned to move critically ill patients between departments? Have these staff received training on identifying and moving critically ill patients? Does this ward have routines or protocols for moving critically ill patients?			
**Person**	Low	Med	High
Have staff in this ward been trained to move critically ill patients between departments? Do you have the required number of staff trained to do this during every shift?			
**Physical environment**	Low	Med	High
When patients are moved is there a space to move them into at the destination department?			
**External Influence**	Low	Med	High
When patients are moved between departments are exceptions made to ensure they continue receiving patients?			
